# FACTORS ASSOCIATED WITH PERCEIVED WORK ABILITY AMONG PEOPLE WORKING ONE YEAR AFTER STROKE: AN EXPLORATORY CROSS-SECTIONAL STUDY

**DOI:** 10.2340/jrm.v58.45817

**Published:** 2026-10-06

**Authors:** Ingrid LINDGREN, Anna NORLANDER, Hélène PESSAH-RASMUSSEN, Christina BROGÅRDH

**Affiliations:** 1 Department of Health Sciences, Lund University, Lund, Sweden; 2 Department of Neurology, Rehabilitation Medicine and Memory Disorders, Skåne University Hospital, Skåne, Sweden; 3 Department of Clinical Sciences, Lund, Lund University, Lund, Sweden

**Keywords:** impairments, return to work, stroke, vocational rehabilitation, work ability

## Abstract

**Objective:**

Knowledge of factors contributing to sustained work ability after stroke is limited. This study explored the association between perceived work ability and personal, stroke-related and work-related factors.

**Design:**

Cross-sectional study.

**Subjects:**

Individuals working 1 year after stroke.

**Methods:**

A postal survey was completed by 88 individuals. The survey comprised questions on demographics, personal, stroke-related and work-related factors, and a set of validated questionnaires including the Work Ability Index (WAI). Data were analysed using logistic regression analyses with perceived overall work ability (WAI item 1) as the dependent variable (good or excellent vs poor or moderate work ability).

**Results:**

Good or excellent work ability was perceived by 59% of the participants. In the final multivariable regression analyses, greater fatigue (OR 0.4; *p* < 0.001) and a poor work–life balance (OR 0.4; *p* < 0.005) were associated with lower odds for perceiving good or excellent work ability, whereas a higher monthly income (OR 5.8; *p* < 0.008) and greater mastery of work (OR 3.2; *p* < 0.007) were associated with higher odds.

**Conclusion:**

Both stroke-related and work-related factors were associated with perceived work ability among individuals working 1 year after stroke. This indicates that several aspects need to be addressed to accomplish a sustainable work situation after stroke.

## LAY ABSTRACT

The aim of this study was to explore the association between perceived work ability and personal, stroke-related and work-related factors among people working 1 year after stroke. A postal survey was completed by 88 individuals. The postal survey comprised questions regarding personal, stroke-related, and work-related factors, including perceived work ability and psychological and social factors at work. The results showed that 59% of the individuals perceived a good or excellent work ability. Greater fatigue and a poor work–life balance was negatively associated with work ability, whereas a higher monthly income and a higher sense of competence and control in relation to the work tasks was positively associated with work ability. The findings show that both stroke-related and work-related factors are associated with perceived work ability among individuals working 1 year after stroke. This suggests that several aspects need to be addressed to accomplish a sustainable work situation after stroke.

Among all people who suffer a stroke in Sweden, about 15–20% are younger than 65 years (i.e., of working age) (1). Of those who worked before the stroke, sick leave for a shorter or longer period after the stroke is common (2). However, many people wish to return to work (3). Being able to return to work after stroke is important for the individual’s health, well-being (4), and financial situation (5, 6). It also reduces the financial burden on the social welfare system (7).

Several factors are important for return to work, such as stroke severity, age, social situation, and characteristics of the work (8, 9). Having a milder stroke (8), white-collar work (10), and higher education (11) increase the possibilities of returning to work. However, it is not only of importance to return to work after stroke, but also to achieve a sustainable work situation in a longer perspective (5). Work is a central part of a person’s life (5), and, with appropriate conditions, people with stroke might be able to contribute to the workplace in the long term (5). In order to achieve a sustainable work situation after stroke, a central component is the person’s own perception of his/her work ability (12).

Work ability can be described as a balance between a person’s resources and the demands of the work (13). It has been found to be associated with physical and psychological function, cognitive, social, and behavioural skills, as well as workplace factors. Important life events, as well as financial and societal issues, seem also to play a role in work ability (14).

In a previous study of various populations, perceived poor work ability has been associated with future sick leave (15). After stroke, many people struggle with persistent problems such as physical impairments, fatigue (16, 17), and concentration difficulties (11, 17), as well as various comorbidities (18, 19). These problems can lead to reduced work ability, which might result in reduced working hours (11, 20, 21) or recurring sick-leave (18, 19). Qualitative studies (17, 22) have described that stroke-related factors such as concentration difficulties, fatigue, emotional difficulties, and muscle weakness can impede the perceived work ability. The importance of work-related factors has also been emphasized, including workplace flexibility and adjustments, flexible work schedules (14, 22, 23), support (17, 22, 23), and work–life balance (23). However, quantitative research that confirms these findings and examines perceived work ability from a broader perspective, including both personal and environmental factors, is lacking.

Thus, more knowledge is needed concerning factors that can contribute to perceived work ability after stroke. Using patient-reported outcomes of work ability can contribute to a better understanding of how healthcare can support people with stroke to find a sustainable work situation. Against this background, the aim of the present study was to explore the association between perceived work ability and personal, stroke-related, and work-related factors among people who had returned to work and were still working 1 year after stroke.

## METHODS

### Study design

This is an exploratory study with a cross-sectional design. It is part of a larger project on returning to work after stroke (11, 16, 23–25). The present study includes data from a postal survey comprising background information, study-specific questions related to return to work, and a set of rating scales on work situation, work ability, self-efficacy, and fatigue. The study was approved by the Regional Ethics Review Board in Lund, Sweden (Dnr 2016/1064), and conducted in accordance with the Helsinki Declaration.

### Participants

A total of 88 individuals who had suffered a stroke 1 year (10–14 months) earlier, had returned to work, and who were still working at the time of the survey, were included in the present study. They were recruited from Skåne University Hospital. Inclusion criteria were: age 18–64 years at stroke onset; admitted to the hospital for acute care due to stroke; referred to the stroke rehabilitation outpatient clinic within 180 days after stroke onset; and worked at least 10 h per week prior to stroke. Individuals who were not sufficiently fluent in Swedish or who had severe cognitive and/or language impairments making them unable to respond to the questionnaire were excluded. Written informed consent was obtained from the participants. A flowchart showing the inclusion of participants is presented in Fig. 1. The recruitment process and data collection are described in more detail elsewhere (11).

**Fig. 1:**
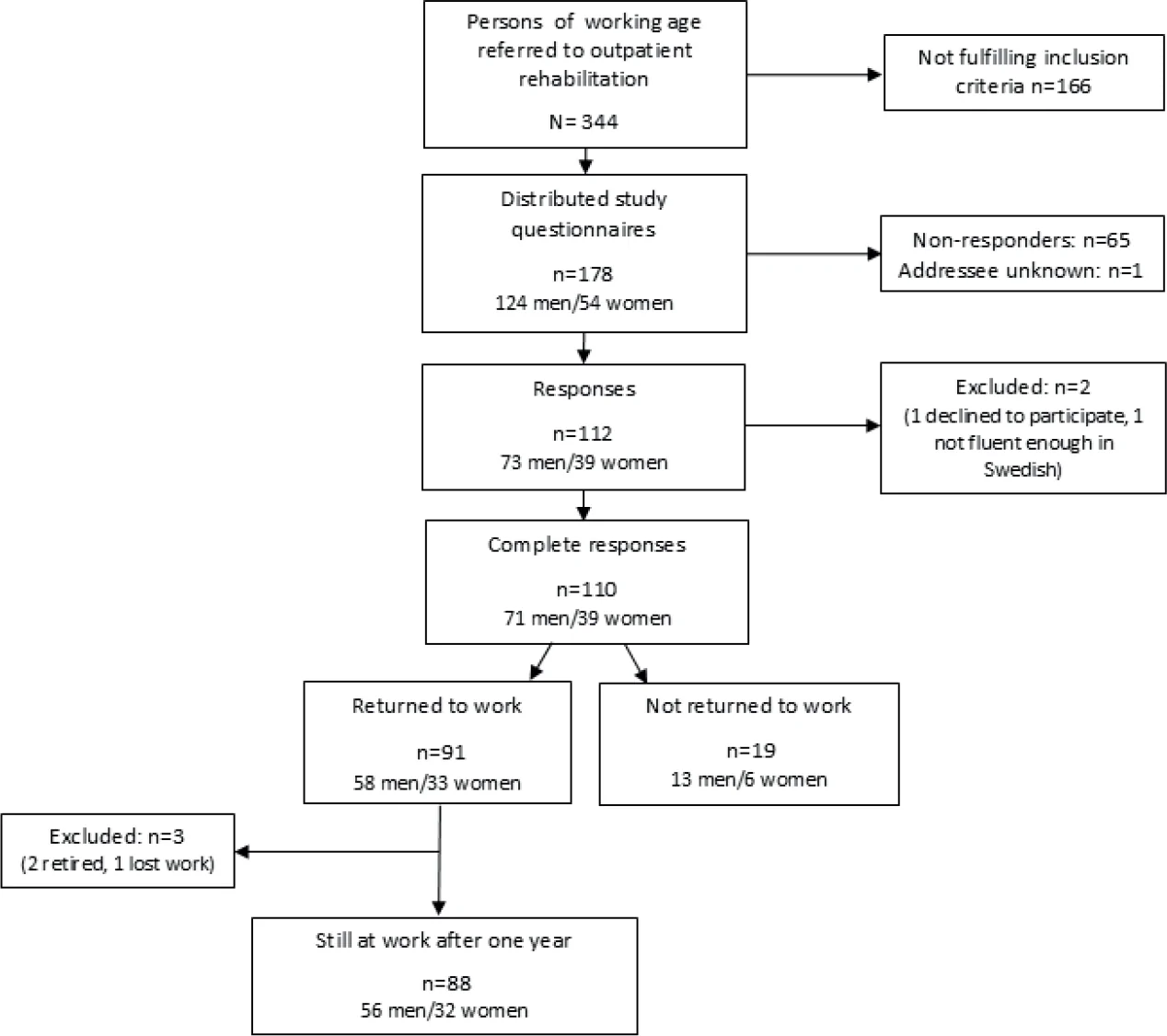
Flowchart of the recruitment process.

### Data collection

*Background variables.* Information regarding stroke type and age was retrieved from medical records. From the postal survey the following variables were retrieved: sex (men/women), walking aids (yes/no), education (elementary school, upper secondary school/high school, college/university), living situation (living alone or together with someone), received rehabilitation focusing on return to work, RTW, (yes/no), size of work organization (up to 49 employees, or 50 or more employees in the organization), employer (private/public), length of current employment (years), monthly income (low < 20,000 SEK, middle 20,000–40,000 SEK, and high, > 40,000 SEK), working hours before and after stroke (0–74%, 75% and above), work characteristics, and type of work (sedentary, mobile, alternating between mobile and sedentary).

### Rating scales

- *Work Ability Index.* The Work Ability Index (WAI) is a questionnaire to assess self-perceived work ability. It is multidimensional and comprises 7 domains (13, 26). WAI item 1, also called the Work Ability Score (WAS), is an indicator of overall work ability (27). The participant is asked to rate his/her current work ability compared with the lifetime best, ranging from 0 (“completely unable to work”) to 10 (“work ability at its best”). The WAS is considered a valid and reliable tool to assess work ability (27).
- *Stroke Impact Scale.* The Stroke Impact Scale (SIS) (28), was used to assess perceived recovery and remaining disabilities after stroke. The SIS includes 8 domains with questions regarding: strength, memory and thinking, mood and emotions, communication, daily activities, mobility, hand function, and participation. A standardized domain-score ranging from 0–100 is calculated for each domain, where 100 indicates no remaining problems. The participant also rated his/her overall recovery (domain 9) on a visual analogue scale ranging from 0 (no recovery) to 100 (full recovery). The SIS is considered valid and reliable, with Cronbach’s alpha ranging from 0.80–0.95 (29). In the present study, the SIS domains memory and thinking, mood and emotions, mobility, hand function, and overall recovery were used.
- *Fatigue Severity Scale.* The Fatigue Severity Scale (FSS) (30), was used to assess perceived impact of fatigue on daily life. In this study, the 9-item Swedish translated version (31) was used. The items are scored on a Likert scale ranging from 1 (strongly disagree) to 7 (strongly agree). The total score is the mean score of the 9 items, where a greater score indicates more fatigue. Good psychometric properties are reported for FSS in people with stroke (32).
- *General Self-Efficacy Scale.* The General Self-Efficacy Scale (GSE) was used to assess the participant’s overall confidence in his/her ability to manage different situations in life. GSE includes 10 statements with 4 response categories ranging from 1 (not at all true) to 4 (exactly true). The total score ranges between 10 and 40, where a higher score represents better self-efficacy. The scale has been found to have good psychometric properties (33).
- *Psychological and social factors at work.* The General Nordic Questionnaire (QPS Nordic) was used to assess psychological and social factors at work (34). It comprises 28 subscales with 2–5 questions in each subscale. In the present study, the following subscales were used: Job demands, Role expectations, Positive expectations, Control at work, Work pace control, Mastery of work (i.e., a person’s sense of competence and control in relation to the work tasks), Social support from manager, Social support from co-workers, Social support from friends and family, and Interaction between work and private life (in this study referred to as “work–life balance”). Each question has 5 response categories, ranging from 1 to 5. In most subscales a higher score is positive, but for the subscales relating to job demands and work–life balance a higher score is negative, i.e., higher strain. The subscale score (range 1–5) is the mean score of the questions in each subscale. QPS Nordic has been shown to be a valid and reliable questionnaire, with Cronbach’s alpha ranging from 0.73–0.84 (35).

### Data analysis

All variables were evaluated and presented using descriptive statistics; *n* (%) or median (interquartile range, IQR). To investigate the association between perceived work ability and potential influencing factors, the Work Ability Score (WAS, i.e., WAI Item 1) was used as the dependent variable in the regression analyses. It was dichotomized into 1–7 (poor or moderate work ability) and 8–10 (good or excellent work ability). The independent variables included personal and stroke-related factors (*n* = 8) as well as work-related factors (*n* = 14) (see Table II). The variables were selected based on previous studies (8, 16, 17, 20, 22) and the authors’ experiences from clinical work with stroke care and rehabilitation. One question in QPS Nordic regarding Control at work, (i.e., “Can you decide when to have contact with clients?”) was not applicable to the work situation for 7 of the participants. Therefore, this question was excluded when calculating the subscale score of these 7 participants. The variable Education was dichotomized to college/university vs upper secondary school or elementary school, and the variable monthly income was dichotomized with a cut-off at 40, 000 SEK.

Univariable logistic regression analyses, with the WAS as the dependent variable, were performed to identify potential independent variables for the multivariable logistic regression analyses. Model validation was performed using Hosmer–Lemeshow goodness of fit (*p* > 0.05). The variable Positive expectations did not fulfil the Hosmer–Lemeshow goodness of fit and was excluded from further analysis. Variables met criteria for multivariable analysis if the *p*-value was < 0.2 in the univariable analysis. Two multivariable regression models were built: 1 for personal and stroke-related factors, and 1 for work-related factors. In each model, 1 independent variable at a time was manually removed, starting with the variable with the highest *p*-value. To prevent overfitting, 4 variables were allowed in each final model. The odds ratio (OR) with 95% confidence interval (CI) and *p*-value were calculated. Nagelkerke’s *R*-squared was used to describe goodness of fit for the final multivariable logistic regression models. The significance level was set to *p* < 0.05. IBM Statistics version 29 (IBM Corp, Armonk, NY, USA) was used in all analyses.

## RESULTS

The survey was sent to 178 individuals, of whom 110 returned complete responses (i.e., a response rate of 62%). Of these, 88 had returned to work and were still working 1 year after stroke (see Fig 1). Seventy (80%) of the participants had had an ischaemic stroke. Most perceived themselves to be well recovered (median [IQR] 90 [77–95]) and few reported problems with mobility (median [IQR] 100 [36–100]) or hand function (median [IQR] 100 [95–100]) on the SIS. Also, no one used walking aids. Time from stroke to return to work was median (IQR) 3 (2–6 months). The participants represented a large variety of professions, both white-collar and blue-collar. The majority (89%) had daytime jobs. The type of work varied, with approximately equal proportions reporting mobile, sedentary, or alternating job tasks. Regarding employment status, 93% reported that they had worked 75–100% full time before their stroke, while 71% did so after stroke. According to WAS, good or excellent work ability was perceived by 59%, and poor or moderate work ability by 41%. Additional descriptive information is presented in Table I.

**Table I:** Descriptive characteristics of the 88 participants

*Personal and stroke-related factors:*	
Age at stroke onset, years, median (IQR)	53 (45–58)
Sex, *n* (%)	
Men	56 (64)
Women	32 (36)
Education completed, *n* (%)^e^	
College/university	35 (40)
Upper secondary school or Elementary school	52 (60)
Living situation, *n* (%)	
Living with someone	70 (79)
Living alone	18 (21)
GSE^a^, median (IQR)^f^	33 (30–36)
FSS^b^, median (IQR)^e^	3.7 (2.3–5)
SIS memory and thinking^c^, median (IQR)^e^	93 (79–100)
SIS mood and emotion^c^, median (IQR)^e^	78 (61–89)
*Work-related factors:*	
Have received RTW rehabilitation, *n* (%)^e^	49 (56)
Size of work organization, *n* (%)^e^	56 (64)
1–49 employees	31 (36)
≥ 50 employees	56 (64)
Employer, *n* (%)^f^	
Private	51 (59)
Public	35 (41)
Years at present workplace, median (IQR)^e^	13 (4–23)
Monthly income, *n* (%)	
Low or middle (≤ 40,000 SEK)	61 (39)
High (> 40,000 SEK)	27 (35)
QPS Nordic subscales:^d^	
Job demands, median (IQR)^e^	2.5 (2.0–3.3)
Role expectations, median (IQR)^e^	4.7 (3.7–5.0)
Positive expectations, median (IQR)^e^	4.3 (3.7–5.0)
Control at work, median (IQR)^e^	3.4 (2.8–4.2)
Work pace control, median (IQR)^e^	3.8 (2.8–4.3)
Mastery of work, median (IQR)^e^	4.3 (4.0–4.8)
Social support from manager, median (IQR)^f^	4.0 (3.0–5.0)
Social support from co-workers, median (IQR)	4.0 (4.0–5.0)
Social support from friends and family, median (IQR)	4–0 (3.3–4.0)
Work-life balance, median (IQR)	2.0 (1.1–2.5)

a GSE, General Self Efficacy Scale 10–40, higher score = better self-efficacy;

b FSS, Fatigue Severity Scale 1–7, higher score = more fatigue;

c SIS, Stroke Impact Scale 0–100 for each domain, higher score = less disability;

d Variables from General Nordic Questionnaire for Psychological and Social factors at Work, subscale score 1–5. On all subscales except “Job demands” and “Work–life balance” (i.e., Interaction between work and private life) a higher score is better; RTW: return to work.

e *n* = 87;

f *n* = 86.

### Factors associated with perceived work ability after stroke

*Personal and stroke-related factors.* In the univariable logistic regression analysis with personal and stroke-related factors, 6 variables had a *p*-value < 0.2 (Table II). The variables were sex, living situation, general self-efficacy (GSE), fatigue (FSS), memory/thinking (SIS item 2), and mood/emotions (SIS item 3). These were included in the multivariable logistic regression analysis. The 4 variables that remained in the final multivariable model were self-efficacy, fatigue, mood/emotions, and living situation (Table III). The only variable that had a significant independent association with perceived work ability was fatigue. More fatigue was associated with lower odds for perceiving a good to excellent work ability (OR, 0.4; *p* < 0.001). The Nagelkerke value for the final multivariable model was 0.505.

**Table II:** Univariable regression analysis with WAS^a^ as the dependent variable

Independent variables	Odds ratio (95% CI)	*p*-value
*Personal and stroke-related factors:*		
Age (years)	0.9 (0.9–1.0)	0.787
Sex (women vs ref. men)	***2.7 (1.1–6.6)***	***0.029***
Education completed (college/university vs ref. upper secondary school or elementary school)	0.7 (0.3–1.7)	0.501
Living situation (living alone vs ref. living with someone)	***2.8 (1.0–8.2)***	***0.056***
GSE^b^ (10–40)	***1.2 (1.1–1.3)***	***< 0.001***
FSS^c^ (1–7)	***0.4 (0.2–0.6)***	***< 0.001***
SIS memory and thinking^d^ (1–5)	***1.1 (1.0–1.1)***	***< 0.001***
SIS mood and emotions^d^ (1–5)	***1.1 (1.0–1.1)***	***< 0.001***
*Work-related factors:*		
Have received RTW rehabilitation, (yes vs ref. no)	0.6 (0.3–1.5)	0.315
Working organization (≥50 vs ref. 1–49 employees)	1.2 (0.5–2.7)	0.809
Employer (public vs ref. private)	***0.5 (0.2–1.1)***	***0.096***
Years at present workplace	1.0 (0.9–1.0)	0.456
Monthly income (high vs ref. low or middle)	***2.6 (1.0–7.0)***	***0.061***
QPS Nordic subscales:^e^		
Job demands (1–5)	0.9 (0.5–1.4)	0.557
Role expectations (1–5)	1.1 (0.6–1.9)	0.758
Control at work (1–5)	***1.5 (1.0–2.5)***	***0.068***
Work pace control (1–5)	1.3 (0.8–2.0)	0.324
Mastery of work (1–5)	***3.3 (1.6–6.9)***	***0.002***
Social support manager (1–5)	1.3 (0.9–2.0)	0.202
Social support from co-workers (1–5)	***1.6 (1.0–2.8)***	***0.063***
Social support from friends and family (1–5)	***2.0 (1.3–3.2)***	***0.003***
Work–life balance (1–5)	***0.5 (0.3–0.8)***	***0.007***

a WAS, Work Ability Scale;

b General Self Efficacy Scale 10–40, higher score means better self-efficacy;

c FSS, Fatigue Severity Scale 1–7, higher score means more fatigue;

d SIS, Stroke Impact Scale, 0–100 for each domain, higher score means less disability;

e Variables from General Nordic Questionnaire for Psychological and Social factors at Work, subscale score 1–5. On all subscales except “Job demands” and “Work–life balance” (i.e., Interaction between work and private life) a higher score is better; ref: reference category in the logistic regression analysis; Variables included in the multivariable analyses are marked in italics and bold.

**Table III:** Final multivariable logistic regression model for personal and stroke-related factors with WAS^a^ as the dependent variable

Independent variables	Odds ratio (95% CI)	*p*-value	Nagelkerke *R*-squared
Living situation (Living alone vs ref living together with someone)	0.3 (1.0–1.5)	0.143	0.505
GSE^b^ (10–40)	1.1 (1.0–1.3)	0.133	
FSS^c^ (1–7)	***0.4 (0.3–0.7)***	***< 0.001***	
SIS mood and emotions^d^	1.0 (1.0–1.1)	0.468	

a WAS, Work Ability Score (Work Ability Index item 1), poor–moderate (1–7), good–excellent (8–10);

b GSE, General Self Efficacy Scale 10–40, higher score means better self-efficacy;

c FSS, Fatigue Severity Scale 1–7, higher score means more fatigue;

d SIS, Stroke Impact Scale, higher score means less disability. Associations with *p* < 0.05 are marked in italics and bold.

*Work-related factors.* In the univariable logistic regression analysis with work-related factors, 7 variables had a *p*-value < 0.2 (see Table II). These variables were: employer, monthly income, and the QPS Nordic variables Control at work, Mastery of work, Social support from co-workers, Social support from friends and family, and Work–life balance. The 4 variables that remained in the final multivariable model were: Monthly income, Mastery of work, Work–life balance, and Social support from family and friends (Table IV). All variables except Social support from friends and family were independently associated with perceived work ability. A poor work–life balance was associated with lower odds for perceiving good to excellent work ability (OR 0.4; *p* < 0.005). Greater mastery of work (OR 3.2; *p* < 0.007) and higher monthly income (OR 5.8; *p* < 0.008) were associated with higher odds for good to excellent work ability (Table IV). The Nagelkerke value for the final multivariable model was 0.406.

**Table IV:** Final multivariable logistic regression model for work-related factors with WAS^a^ as the dependent variable

Independent variables	Odds ratio (95% CI)	*p*-value	Nagelkerke *R*-squared
Monthly income^b^ (high vs ref. low–middle)	***5.8 (1.6–21.4)***	***0.008***	0.406
Mastery of work^c^ QPS N subscale (1–5)	***3.2 (1.4–7.3)***	***0.007***	
Social support from friends and family^c^ QPS N subscale (1–5)	1.6 (1.0–2.7)	0.057	
Work–life balance^d^ QPS N subscale (1–5)	***0.4 (0.2–0.7)***	***0.005***	

a Dependent variable: WAS, Work Ability Score (Work Ability Index Item 1), poor-moderate (1–7), good–excellent (8–10);

b monthly income dichotomized low–middle income vs high income;

c variables from General Nordic Questionnaire for Psychological and Social factors at Work, QPS N. On all subscales except “Work-life balance” (i.e., Interaction between work and private life) a higher score is better. Associations with *p* <0.05 are marked in italics and bold.

## DISCUSSION

This study aimed to explore the association between perceived work ability and personal and stroke-related factors as well as work-related factors among people who had returned to work and were working 1 year after stroke. The results showed that 59% of the participants perceived good or excellent work ability. Although several personal and stroke-related factors were related to work ability in the univariable analyses, only fatigue was independently associated with perceived work ability. Among work-related factors, a poor work–life balance was associated with lower odds for perceiving good or excellent work ability, whereas a higher monthly income and greater mastery of work were associated with higher odds.

*Personal and stroke-related factors.* Our results demonstrate that higher scores on the FSS (i.e., more fatigue) were associated with lower odds for perceiving a good to excellent work ability. Fatigue can be defined as “a feeling of exhaustion, weariness or lack of energy that can be overwhelming, and which can involve physical, emotional, cognitive and perceptual contributors, which are not relieved by rest and affects a person’s daily life” (36). It has previously been reported to affect between 25% and 85% of stroke survivors during the first 2 years after stroke (37) and has been shown to be a significant problem for many people who return to work after stroke (14, 17, 22, 25). Even if the participants in our study had no or minor sensorimotor impairments and considered themselves well recovered (SIS-9 median 90%), about 40% reported problems with fatigue on the FSS (total score ≥ 4). Currently, there are no established medical treatments for fatigue. However, qualitative studies (6, 11) have shown that support and adjustments in the work situation can mitigate fatigue and other invisible stroke impairments. Support and adjustments can include adapting the work schedule, enabling pauses, noise reduction, and lessening other disrupting impressions in the work environment (6, 11). To optimize individual adjustments in the work situation might be a key factor for a sustainable work situation. However, to be able to stay at work in a longer perspective after stroke, recurrent adjustments and follow-ups might be needed (17). Involvement might be needed not only from the employee and supervisor, but also from other stakeholders and rehabilitation professionals.

Moreover, self-efficacy, memory/thinking, mood/emotions, and social support from family and friends did not remain significant in our multivariable models. In previous studies, self-efficacy (36), cognition (17), mood (8), and social support (5, 17, 22) have been described as being important for returning to work as well as for work ability.

*Work-related factors.* In our multivariable analysis, 3 work-related factors were independently associated with perceived work ability. First, a poor work–life balance was associated with lower odds for perceiving good to excellent work ability. The questions asked in the survey were related to whether work interfered in a negative way with private life, or whether private life interfered negatively with work. Given that many of our participants suffered from fatigue, the result seems reasonable. In qualitative studies, individuals with fatigue after stroke have described difficulties prioritizing activities (17) and having to reduce social activities (38). Thus, achieving a sustainable work situation may require more than workplace adjustments alone. Individuals may need professional support to address their overall life circumstances, as well as any persistent stroke-related impairments, in order to establish a better work–life balance.

Moreover, the analysis in our study also revealed that a higher income was related to higher perceived work ability, which may reflect greater access to external support or other socioeconomic resources in private life. The odds for perceiving a good to excellent work ability were more than 5 times as high for those with a high income compared with those with a low–middle income. Income has previously been shown to be an important factor for returning to work (6) as well as for maintaining work (14). Income of course enables the individual to contribute economically to the household (10), which might give self-confidence. Also, white-collar work, which is commonly better paid, has been found to be a predictor for return to work (10). This might be related to the type of work tasks and more flexible work situation.

Another finding in our study was that high mastery of work was associated with higher odds for perceiving a good to excellent work ability. Being able to contribute to the work is positive for the individual and enhances self-confidence (5). For a sustainable work situation, it may be important to adapt work duties to the individual’s work ability, allowing the individual to experience a stronger sense of competence and contribution.

### Strengths and limitations

A strength of this study is its focus on self-perceived work ability in a longer perspective after returning to work after stroke. This is a topic that has received limited attention in previous studies. In addition, a broad range of potential influencing factors was evaluated, encompassing personal, stroke-related, and work-related factors. However, the study also has some limitations. The exploratory cross-sectional design limits the possibility of drawing any causal conclusions, and the relatively small sample size limits the number of variables that could be included in the multivariate analysis. The 3 individuals who had stopped working within a year (due to retirement or other reasons) were not included in our analysis as they did not fulfil the inclusion criteria. The reasons why individuals who have returned to work after stroke choose to stop working need to be acknowledged in future studies.

The participants in the present study were identified through an outpatient stroke rehabilitation clinic; several had received various modes of support or adjustments at their workplaces, and most considered themselves to be fairly well recovered from their stroke. Still, given that more people experience mild disabilities after a stroke, our findings are important and indicate that several factors should be considered to ensure a sustainable work situation. Future studies should include a broader stroke population and also those who have recently stopped working, and investigate how access to external support influences work ability, as well as how work ability is perceived over a longer period following a stroke.

To summarize, achieving a sustainable work situation after stroke is important, as it benefits both the individual and society. Personal and stroke-related factors as well as work-related factors should be considered to enable individualized workplace adjustments and support, ultimately promoting sustained work ability after stroke.

In conclusion, the results from this study show that both stroke-related and work-related factors are associated with perceived work ability among individuals working 1 year after stroke. The multifaced picture of factors included perceived fatigue, the individual’s sense of competence and control in the work situation, and work–life balance. Thus, several aspects need to be considered and followed up to accomplish a sustainable work situation after stroke.
